# Comprehensive Mapping Antigenic Epitopes of NS1 Protein of Japanese Encephalitis Virus with Monoclonal Antibodies

**DOI:** 10.1371/journal.pone.0067553

**Published:** 2013-06-18

**Authors:** Rong-Hong Hua, Li-Ke Liu, Zhen-Shi Chen, Ye-Nan Li, Zhi-Gao Bu

**Affiliations:** State Key Laboratory of Veterinary Biotechnology, Harbin Veterinary Research Institute, Chinese Academy of Agricultural Sciences, Harbin, Heilongjiang, People's Republic of China; Thomas Jefferson University, United States of America

## Abstract

Japanese encephalitis virus (JEV) non-structural protein 1 (NS1) contributes to virus replication and elicits protective immune responses during infection. JEV NS1-specific antibody responses could be a target in the differential diagnosis of different flavivirus infections. However, the epitopes on JEV NS1 are poorly characterized. The present study describes the full mapping of linear B-cell epitopes in JEV NS1. We generated eleven NS1-specific monoclonal antibodies from mice immunized with recombinant NS1. For epitope mapping of monoclonal antibodies, a set of 51 partially-overlapping peptides covering the entire NS1 protein were expressed with a GST-tag and then screened using monoclonal antibodies. Through enzyme-linked immunosorbent assay (ELISA), five linear epitope-containing peptides were identified. By sequentially removing amino acid residues from the carboxy and amino terminal of peptides, the minimal units of the five linear epitopes were identified and confirmed using monoclonal antibodies. Five linear epitopes are located in amino acids residues ^5^AIDITRK^11^, ^72^RDELNVL^78^, ^251^KSKHNRREGY^260^, ^269^DENGIVLD^276^, and ^341^DETTLVRS^348^. Furthermore, it was found that the epitopes are highly conserved among JEV strains through sequence alignment. Notably, none of the homologous regions on NS1 proteins from other flaviviruses reacted with the MAbs when they were tested for cross-reactivity, and all five epitope peptides were not recognized by sera against West Nile virus or Dengue virus. These novel virus-specific linear B-cell epitopes of JEV NS1 would benefit the development of new vaccines and diagnostic assays.

## Introduction

Japanese encephalitis (JE) is caused by the Japanese encephalitis virus (JEV), and is one of the most important mosquito-borne diseases with a mortality rate as high as 20% to 50%, and is widely distributed in most of East and South-east Asia and parts of Oceania. Up to 50,000 human cases of JE are reported annually in Asian countries, of which 10,000–15,000 result in fatality [1]. A high proportion (nearly 50%) of survivors, especially young children and those greater than 65 years of age, exhibit permanent neurologic and psychiatric sequelae. A wide range of animals including swine, equines and birds can also be infected. Pigs, as well as birds, serve as amplifying and reservoir hosts [2], [3]. Further, JEV infection has accounted for significant economic losses in the pig industry due to fetal encephalitis and reproductive failure in pregnant sows and hypospermia in boars [4]. There is no specific treatment available for JE, and vaccination is the only effective way to prevent JEV infection in humans and domestic animals. JEV non-structural protein 1 (NS1) has been shown to induce both humoral and cell-mediated immunity against JE [5], [6]. Further, like other flaviviruses, NS1 is able to elicit protective immunity without the risk of antibody-dependent enhancement. These characteristics make NS1 an attractive alternative immunogen. As such, much research is currently being devoted to NS1-based vaccine development [7], [8], [9]. Although NS1 is not present in the virion, NS1-induced antibodies can protect against infection *in vivo* by an undetermined mechanism, which presumably depends on the Fc portion of the antibody since they kill their target cells through a complement-dependent pathway [10], [11].

JEV is a member of the family *Flaviviridae*, and the genus *Flavivirus*, and is primarily transmitted by *Culex* mosquitoes. Besides JEV, the Japanese encephalitis virus serocomplex of *Flaviviridae* includes the West Nile virus (WNV), Saint Louis encephalitis virus (SLEV) and Murray Valley encephalitis virus (MVEM). JEV serogroup viruses and Dengue virus (DENV) have a similar ecology; it is very common that two or more of these flaviviruses co-circulate in some regions of the world [12]–[15], and cross-reactivity can be demonstrated among these flaviviruses in serological tests. These cross-reactive responses could confound the interpretation of results during serological testing, including neutralization tests and enzyme-linked immunosorbent assays (ELISA) [16]. This serves to emphasize the utility of virus-specific epitopes for the differential diagnosis of disease and epidemiological surveys. The serological cross-reactivity is primarily caused by cross-reactive epitopes on the structural protein E [17], [18]. In contrast, NS1 is more specific in serological testing of flavivirus infections, and it has been reported that NS1 can induce antibodies without cross-reactivity among flaviviruses [14], and even among different serotypes of DENV [19], [20], therefore the development of an NS1-based specific serological diagnosis is of great interest [14], [21], [22]. First, it is necessary to precisely identify the B-cell epitopes on NS1. In this study we have identified and characterized five JEV NS1-specific epitopes with monoclonal antibodies. This work demonstrates progress toward the development of a specific serological diagnostic test for JEV infection, extends our understanding of the antigenic structure of JEV NS1, and could help inform vaccine design.

## Materials and Methods

### Ethics statement

Care of laboratory animals and animal experimentation was performed in accordance with animal ethics guidelines and approved protocols. All animal experiments were approved by the Animal Ethics Committee of Harbin Veterinary Research Institute of the Chinese Academy of Agricultural Sciences. The Animal Ethics Committee approval number was SYXK (Hei) 2011–022.

### Cell lines, virus and serum

Myeloma cell line SP2/0 and baby hamster kidney BHK-21 cells (CCL-10, American Type Culture Collection) were cultured in RPMI-1640 (Gibco, Grand Island, NY, USA) and Dulbecco's modified Eagle's medium (DMEM; Gibco, Invitrogen, Carlsbad, CA, USA), respectively, in a humidified 5% CO_2_ atmosphere at 37°C. All culture media were supplemented with 10% heat-inactivated fetal bovine serum (FBS; Gibco), 100 U/ml penicillin and 100 µg/ml streptomycin (Gibco). The JEV SA14-14-2 strain (GenBank: AF315119) was propagated and titrated on BHK-21 cell monolayers. Inactivated WNV, DENV-1, DENV-2, DENV-3 and DENV-4 cell cultures were kindly provided by Dr. Cheng-Feng Qin (Beijing Institute of Microbiology and Epidemiology, Beijing, China). The mouse sera against WNV and DENV were also provided by Dr. Cheng-Feng Qin.

### Expression of recombinant NS1

The full-length gene of NS1 was PCR amplified from the plasmid pET30-JEV-NS1 [23]. The sequence of the upstream primer is 5′CGC*CCATG*GACACTGGATGTGCCATTGAC 3′, and that of the downstream primer is 5′TTA*GGATCC*AGCAGCGACTAGCACCACATACC 3′. Primers were designed according to the sequence of JEV strain SA14-14-2 and contain *Nco* I and *Bam*H I restriction enzyme site respectively. After agar gel purification and restriction enzyme digestion, the PCR products were cloned into the expression vector pMAL-c5x (NEB, Ipswich, MA, USA). The resulting recombinant plasmid was verified by enzyme digestion and DNA sequencing. Confirmed recombinant plasmids were transformed into *E. coli* ER2523 (NEB Express, MA, USA) cells for expression. After 1 hour of shaking, isopropyl-D-thiogalactopyranoside (IPTG; Sigma, MO, USA) was added to bacterial cultures in Luria-Bertani broth at a final concentration of 0.3 mM and the cultures were shaken for a further 3 h. Then the bacterial cells were pelleted at 5,000 g for 15 min and lysed by sonication in phosphate buffered saline (PBS), and sodium dodecyl sulfate-polyacrylamide gel electrophoresis (SDS-PAGE) was carried out to analyze the recombinant protein. The recombinant protein MBP-NS1 was purified by MBPTrap HP column affinity chromatography according to the manufacturer's instructions (GE Healthcare Bio-Sciences, MA, USA).

### Preparation of monoclonal antibodies (MAbs) against NS1

The purified recombinant protein was used as an immunogen in mice. Hybridomas secreting anti-NS1 antibodies were generated according to the procedure described previously [24]. Six-week-old female BALB/c mice were immunized subcutaneously with purified MBP-NS1 protein emulsified with an equal volume of Freund's complete adjuvant (Sigma, St. Louis, MO, USA). Two booster injections were given at 2-week intervals with the same immunogen and equal volume of Freund's incomplete adjuvant. The soluble recombinant protein was intraperitoneally injected into the mice without adjuvant as a final immunization. Three days after final administration, the mice were euthanized humanly; spleen cells were harvested and subsequently fused with SP2/0 myeloma cells at 5∶1 ratio using polyethylene glycol (PEG 4000, Sigma). The hybridoma cells were seeded into ninety-six well plates and selected in HAT medium (RPMI-1640 medium containing 10% fetal bovine serum, 100 µg/ml streptomycin, 100 IU/ml penicillin, 100 mM hypoxanthine, 16 mM thymidine and 400 mM aminopterin). On days 3, 6 and 9, half of this medium was removed and replaced with fresh medium containing HAT. After HAT/HT medium selection, culture supernatants of surviving clones were screened for antibody reactivity and specificity by indirect enzyme-linked immunosorbent assay (ELISA) and Western blotting. The positive cell clones were subcloned three times by limiting dilution method. Selected clones were cultured in the peritoneal cavities of pristane-primed BALB/c mice to obtain ascitic fluid.

The identity of the heavy and light chains of each MAb was determined using Pierce Rapid Isotyping Kit with Kappa and Lambda Mouse (Thermo, Rockford, IL, USA).

### Expression of short peptide fusion proteins

In order to map the epitopes of monoclonal antibodies, a set of 51 16-amino acid partially-overlapping short peptides (NS1-1 to NS1-51; Table 1) covering the entire NS1 protein were designed and expressed as a fusion protein with GST in pGEX-6p-1 plasmids as described previously [23]. In fine epitope mapping and specificity identification, all short peptides were expressed as the same way. All GST fusion proteins were purified by Glutathione Sepharose 4B RediPack column affinity chromatography according the manufacturer's instructions (Amersham Pharmacia Biotech).

**Table 1:** Sequences of the overlapping peptides derived from JEV NS1 (SA14-14-2 strain, GenBank AF315119).

Peptide no.	Amino acid sequence	Peptide no.	Amino acid sequence
NS1-1	^1^DTGCAIDITRKEMRCG^16^	NS1-27	209TWKLERAVFGEVKSCT^224^
NS1-2	^9^TRKEMRCGSGIFVHND^24^	NS1-28	217FGEVKSCTWPETHTLW^232^
NS1-3	^17^SGIFVHNDVEAWVDRY^32^	NS1-29	^225^WPETHTLWGDDVEESE^240^
NS1-4	^25^VEAWVDRYKYLPETPR^40^	NS1-30	^233^GDDVEESELIIPHTIA^248^
NS1-5	^33^KYLPETPRSLAKIVHK^48^	NS1-31	^241^LIIPHTIAGPKSKHNR^256^
NS1-6	^41^SLAKIVHKAHKEGVCG^56^	NS1-32	^249^GPKSKHNRREGYKTQN^264^
NS1-7	^49^AHKEGVCGVRSVTRLE^64^	NS1-33	^257^REGYKTQNQGPWDENG^272^
NS1-8	^57^VRSVTRLEHQMWEAVR^72^	NS1-34	^265^QGPWDENGIVLDFDYC^280^
NS1-9	^65^HQMWEAVRDELNVLLK^80^	NS1-35	^273^IVLDFDYCPGTKVTIT^288^
NS1-10	^73^DELNVLLKENAVDLSV^88^	NS1-36	^281^PGTKVTITEDCSKRGP^296^
NS1-11	^81^ENAVDLSVVVNKPVGR^96^	NS1-37	^289^EDCSKRGPSVRTTTDS^304^
NS1-12	^89^VVNKPVGRYRSAPKRL^104^	NS1-38	^297^SVRTTTDSGKLITDWC^312^
NS1-13	^97^YRSAPKRLSMTQEKFE^112^	NS1-39	^305^GKLITDWCCRSCSLPP^320^
NS1-14	^105^SMTQEKFEMGWKAWGK^120^	NS1-40	^313^CRSCSLPPLRFRTENG^328^
NS1-15	^113^MGWKAWGKSILFAPEL^128^	NS1-41	^321^LRFRTENGCWYGMEIR^336^
NS1-16	^121^SILFAPELANSTFVVD^136^	NS1-42	^329^CWYGMEIRPVMHDETT^344^
NS1-17	^129^ANSTFVVDGPETKECP^144^	NS1-43	^337^PVMHDETTLVRSQVHA^352^
NS1-18	^137^GPETKECPDEHRAWNS^152^	NS1-44	^345^LVRSQVHAFKGEMVDP^360^
NS1-19	^145^DEHRAWNSMQIEDFGF^160^	NS1-45	^353^FKGEMVDPFQLGLLVM^368^
NS1-20	^153^MQIEDFGFGITSTRVW^168^	NS1-46	^361^FQLGLLVMFLATQEVL^376^
NS1-21	^161^GITSTRVWLKIREEST^176^	NS1-47	^369^FLATQEVLRKRWTARL^384^
NS1-22	^169^LKIREESTDECDGAII^184^	NS1-48	^377^RKRWTARLTIPAVLGV^392^
NS1-23	^177^DECDGAIIGTAVKGHV^192^	NS1-49	^385^TIPAVLGVLLVLMLGG^400^
NS1-24	^185^GTAVKGHVAVHSDLSY^200^	NS1-50	^393^LLVLMLGGITYTDLAR^408^
NS1-25	^193^AVHSDLSYWIESRYND^208^	NS1-51	^401^ITYTDLARYVVLVAA^415^
NS1-26	^201^WIESRYNDTWKLERAV^216^		

### Enzyme-linked immunosorbent assay (ELISA)

Ninety-six well microtiter plates were coated with purified fusion protein in 0.1 M carbonate buffer (pH 9.6) at 4°C overnight and blocked with 5% skimmed milk for 2 h at 37°C. After washing three times with PBST (PBS plus 0.5% Tween-20), 100 µL of hybridoma supernatant containing MAbs was added to each well and incubated at 37°C for 1 h, followed by three washes with PBST. The bound MAbs were detected with horseradish peroxidase (HRP)-conjugated goat anti-mouse IgG (Zhongshan Biotechnology Co., Ltd Beijing, China). After a further incubation and thorough washing with PBST, 100 µL of 3,3′–5,5′-tetramethyl benzidine (TMB) was added as a substrate for HRP. The reaction was stopped after 15 min with 2 M H_2_SO_4_ and the absorbance was measured at 450 nm by an ELISA plate reader (Bio-Rad, Hercules, CA, USA). Glutathione *S*-transferase (GST) and purified MBP-NS1 were used as negative and positive controls, respectively. Results depict the average of duplicate assays.

### Western blotting

Virus-infected cell lysates or expressed fusion proteins were subjected to electrophoresis on 12% SDS-PAGE after reduction with dithiothreitol (DTT) at 100°C for 5 min, then the samples were transferred to a nitrocellulose membrane. Nonspecific antibody binding sites were blocked with 5% skimmed milk powder in PBS overnight at 4°C. The membranes were incubated with MAbs as the primary antibody, after which each blot was washed five times with PBST and treated with IRDye 700-conjugated goat anti-mouse secondary antibodies (LICOR Biosciences, NE, USA). Blots were visualized by scanning the membranes with a LICOR Odyssey infrared image system (LICOR Biosciences).

## Results

### Production of MAbs

**Figure 1:**
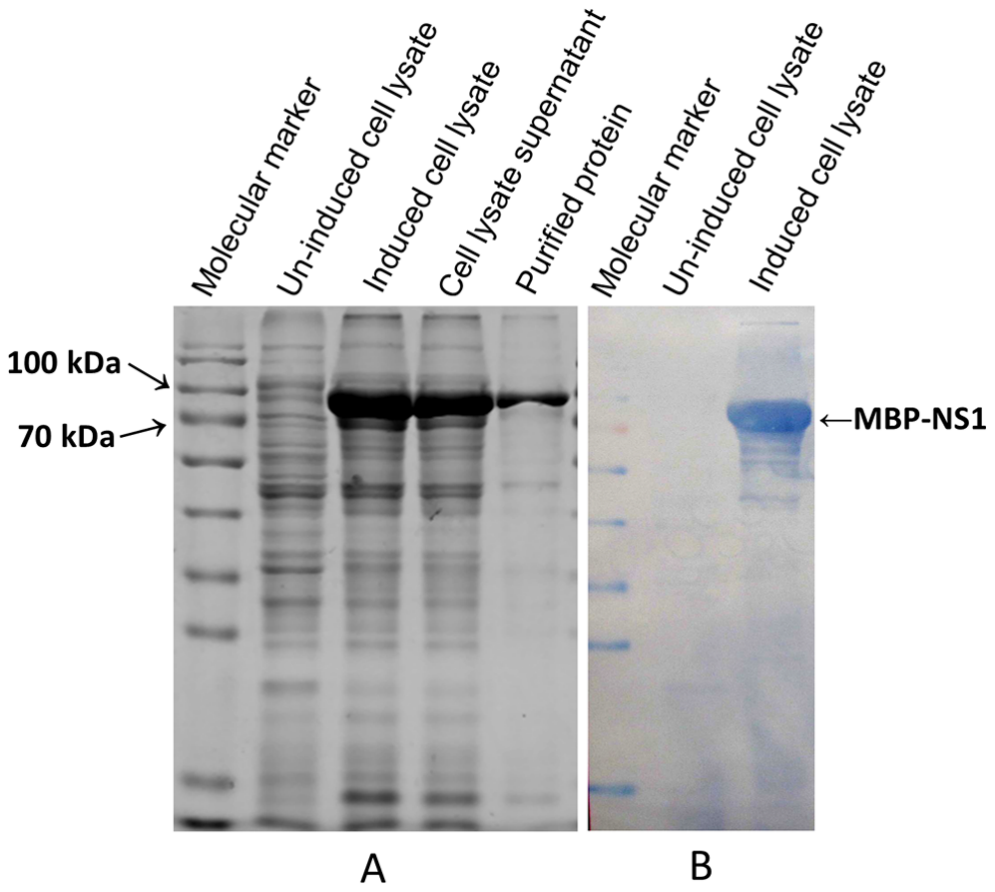
SDS-PAGE and Western blotting analysis of JEV NS1expression. A, SDS-PAGE analysis of recombinant MBP-NS1 protein. MBP-NS1 is approximately 90 kDa. B, Western blotting analysis of recombinant NS1 protein. The MBP-NS1 band was visualized with TMB membrane substrate.

Recombinant JEV NS1 was successfully expressed in *E. coli* ER2523 after induction with IPTG. Most of the expressed fusion protein MBP-NS1 was soluble in the supernatant of the sonified bacterial cell lysate. The recombinant protein could be recognized by JEV-positive serum from mice (Fig. 1), so purified proteins were used to immunize BALB/c mice. After cell fusion and selection, we generated and selected eleven hybridoma cell lines, which produced MAbs that strongly bound recombinant MBP-NS1 and native NS1 of JEV by indirect ELISA and Western blotting. We further analyzed these MAbs for cross-reactivity with WNV, DENV1, DENV2, DENV3 and DENV4 by Western blotting. All the MAbs were JEV specific and did not recognize NS1 from WNV or DENV (Table 2). As shown in Table 2, the NS1-specific MAb heavy-chain subclasses were identified as IgG1, IgG2a or IgG2b, while the light chains were all subtype κ. The titers of antibodies secreted into the culture supernatant and ascitic fluid were measured by indirect ELISA. Antibody titers ranged from 32 to 2048. Antibody titers of ascitic fluid were 204,800 or higher except for 8B5 which was 25,600 (Table 2).

**Table 2:** Characteristics of MAbs against JEV NS1.

MAb	Heavy chain	Light chain	ELISA titre^a^		Western blotting^b^					
			Ascites	Culture supernatant	JEV	WNV	DENV-1	DENV-2	DENV-3	DENV-4
3G11	IgG2a	κ	>204800	2048	+	−	−	−	−	−
3H11	IgG2a	κ	>204800	2048	+	−	−	−	−	−
4E3	IgG1	κ	>204800	64	+	−	−	−	−	−
4E8	IgG1	κ	204800	512	+	−	−	−	−	−
5C6	IgG2a	κ	204800	2048	+	−	−	−	−	−
6D8	IgG2b	κ	>204800	2048	+	−	−	−	−	−
7C11	IgG1	κ	>204800	1024	+	−	−	−	−	−
7G1	IgG1	κ	>204800	1024	+	−	−	−	−	−
8B5	IgG1	κ	25600	32	+	−	−	−	−	−
10F7	IgG2b	κ	204800	256	+	−	−	−	−	−
HA12	IgG1	κ	204800	256	+	−	−	−	−	−

a, The recombinant MBP-NS1 fusion protein was used as antigen in MAbs titration. The titres are expressed as the reciprocal of the highest dilution. b, Virus infected cell lysates are used as antigen in the Western blotting assay. +, Positive reaction with the corresponding virus. −, Negative reaction with the corresponding virus.

### Mapping of antigenic epitopes on JEV NS1

**Figure 2:**
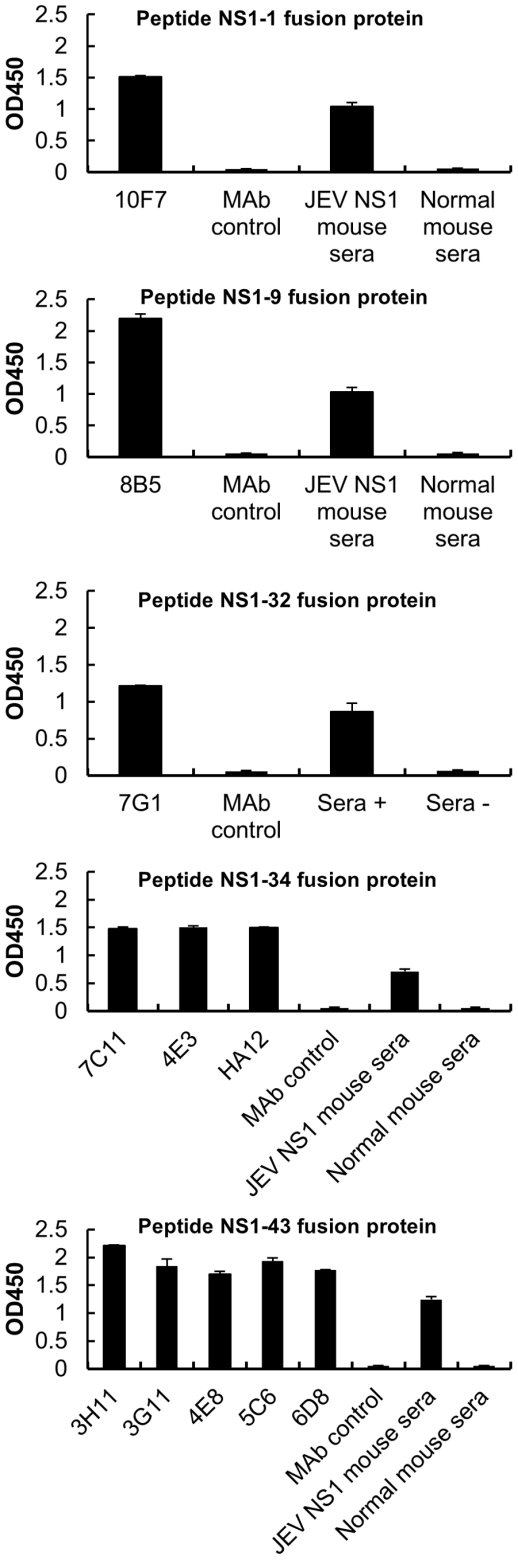
Primary identification of epitopes with MAbs. Five epitopes of JEV NS1 protein were mapped with MAbs by ELISA. Cell culture medium was used as the MAb control. Mouse anti-sera against JEV NS1 and normal mouse sera were used as positive and negative controls, respectively. Each bar indicates antibody reactivity as determined by the mean absorbance.

To map the antigenic epitopes of JEV NS1, 51 short peptides were expressed in a fusion protein with GST and probed by MAbs. ELISA results showed that five peptide fusion proteins were recognized by eleven MAbs. NS1-1, NS1-9 and NS1-32 were recognized by MAb 10F7, 8B5 and 7G1, respectively. NS1-34 was recognized by three MAbs (7C11, 4E3 and HA12), and NS1-43 was recognized by five MAbs (3H11, 3G11, 4E8, 5C6 and 6D8) (Fig. 2). The results indicate that there are at least five linear epitopes located throughout NS1.

### Fine mapping of epitopes

**Figure 3:**
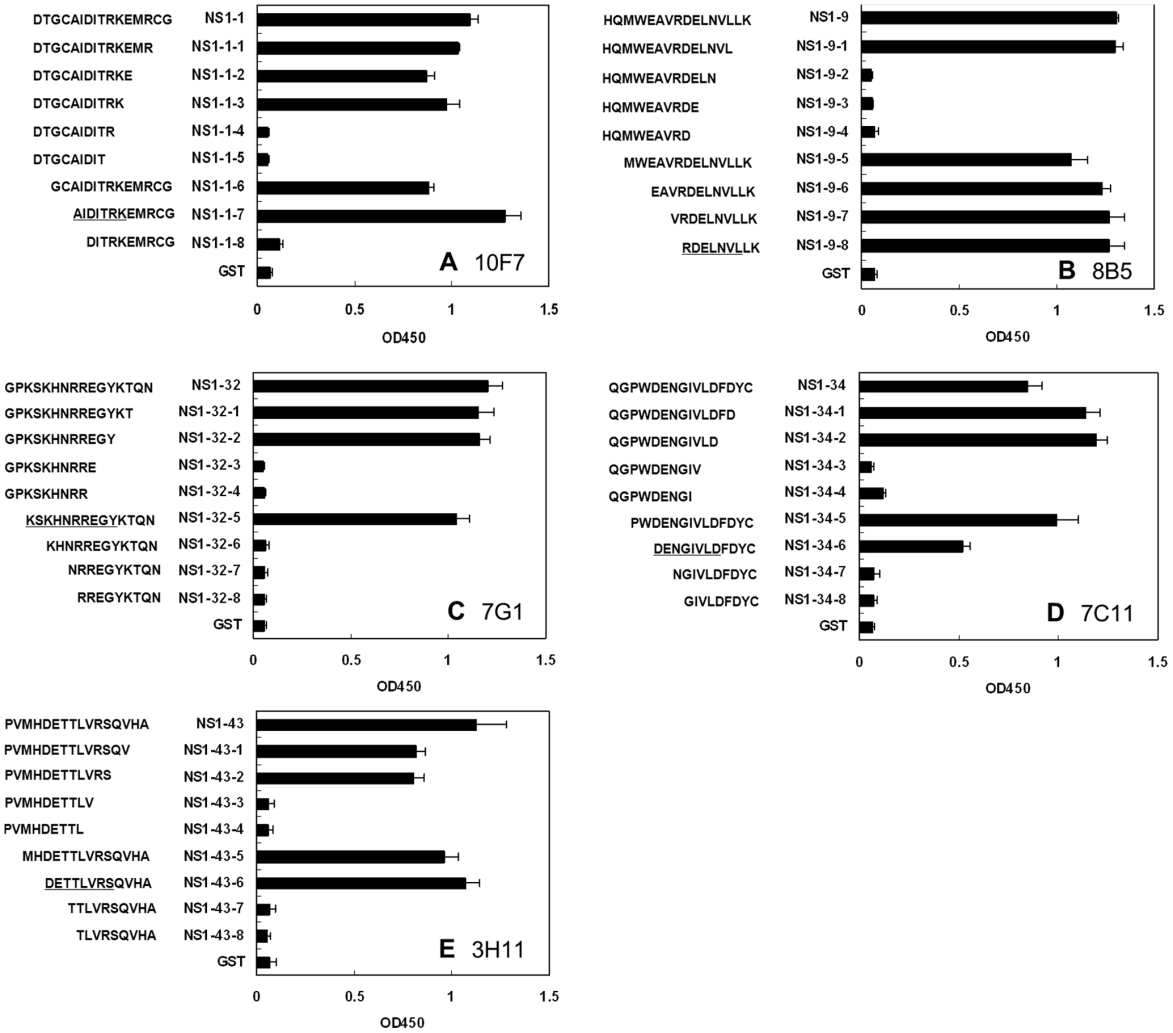
Fine-mapping of the epitopes by ELISA with MAbs. Epitope-containing peptides were truncated from the carboxy and amino terminals. After the truncated peptides were expressed as a GST fusion protein, they were probed with MAbs by ELISA respectively. The deduced minimal unit of each linear B-cell epitope is underlined.

To precisely locate the core sequences of epitopes on NS1, a series of truncated peptides were designed and expressed as a fusion with GST. Firstly, one or two amino acid residues were sequentially removed from the carboxy terminal of the peptide until the minimum peptide was 9 amino acids long. The shortened GST-fusion peptides were analyzed by ELISA using specific MAbs. Next, one or two amino acid residues were removed from the amino terminal of the peptide in the same fashion. The shortened GST-fusion peptides were also analyzed by ELISA with specific MAbs. As Figure 3 indicates, ^5^AIDITRK^11^ is the minimal requirement for MAb 10F7 to recognize the linearized epitope NS1-1. The minimal linear sequence of epitope NS1-9 for MAb 8B5 is ^72^RDELNVL^78^; ^251^KSKHNRREGY^260^ for epitope NS1-32 for MAb 7G1; ^269^DENGIVLD^276^ for epitope NS1-34 for MAb 7C11 (and MAbs 4E3 and HA12 [data not shown]); ^341^DETTLVRS^348^ for epitope NS1-43 for MAb 3H11, 3G11, 4E8, 5C6 and 6D8.

### Identification of conserved and virus specific B-cell epitopes on NS1 of JEV

To investigate whether the identified linear epitopes were specific or conserved among viruses of the JEV isolates, NS1 sequences of SA14-14-2 strain were analyzed by Blast in the NCBI Entre protein database (http://blast.ncbi.nlm.nih.gov/Blast.cgi). From this search, 100 JEV NS1 protein or genome polyprotein sequences were selected for sequence alignment. Alignment of the five linear epitopes of NS1 from different JEV isolates revealed all five epitopes are highly conserved among JEV isolates. Especially the epitopes NS1-9 ^72^RDELNVL^78^ and NS1-34 ^269^DENGIVLD^276^, as 99 of 100 isolates show 100% sequence identity. In the corresponding sequences of all other JEV isolates there is only one amino acid substitution in conserved epitopes (Table 3).

**Table 3:** Sequence alignment of the defined epitopes corresponding to NS1 amino acid residues in JEV isolates.^a^

Peptide name	Sequence	Location^b^	Conserved virus strains/all virus isolates
EP NS1-1	AIDITRK	5–11	88/100
	AIDITRR		7/100
	AIDVTRK		1/100
	AIDIARK		2/100
	AIDTTRK		2/100
EP NS1-9	RDELNVL	72–78	99/100
	RDELNVP		1/100
EP NS1-32	KSKHNRREGY	251–260	86/100
	RSKHNRREGY		13/100
	KSKHNQREGY		1/100
EP NS1-34	DENGIVLD	269–276	99/100
	DEKGIVLD		1/100
EP NS1-43	DETTLVRS	341–348	91/100
	DEATLVRS		9/100

a The JEV NS1 protein sequences or genome polyprotein sequences in this alignment were obtained from the NCBI Entrez Protein database. Sequence blast and alignment were conducted with the online program (http://blast.ncbi.nlm.nih.gov/Blast.cgi).

b The location of the amino acid residues on the JEV NS1 protein.

**Figure 4:**
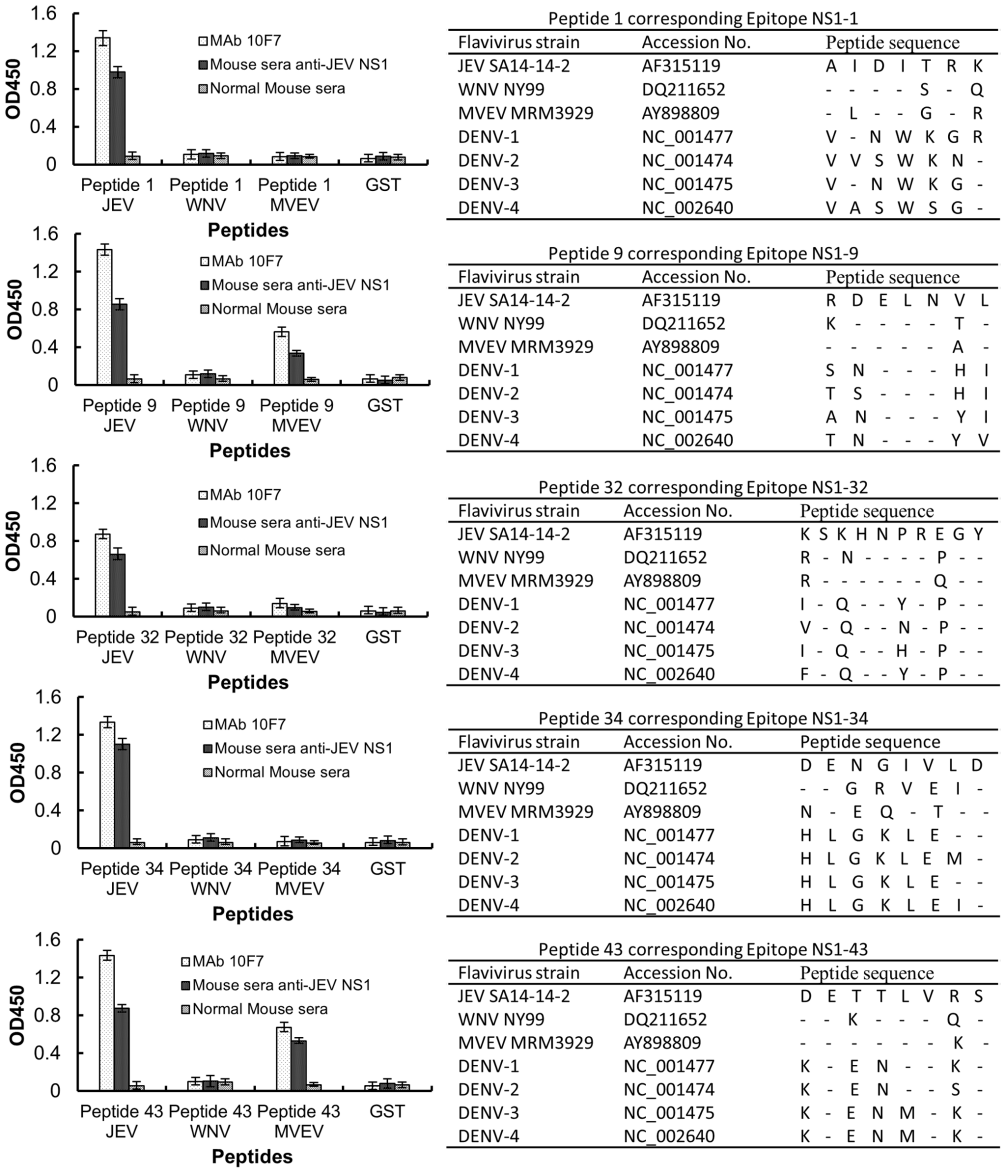
Identification of virus-specific epitopes with MAbs. Sequences were aligned between the identified linear epitopes and the homologous regions of WNV, MVEV and DENV (1–4). The virus strains listed in this figure were selected as representative strains. The homologous peptides from WNV and MVEV epitopes were expressed and analyzed by ELISA using the correspond MAbs. All MAbs and anti-sera against JEV NS1 did not cross-react with the homologous peptides of WNV. For MVEV, only peptides 9 and 43 showed weak reactivity with MAb 8B5 and 3H11.

Sequence alignment was also performed between these five B-cell epitopes with homologous sequences from other flaviviruses including WNV, MVEV and DENV (Fig. 4, right panels). Alignment results showed that all five identified epitopes have less than 50% identity with DENV (1–4) NS1 sequences. After the corresponding peptides for epitopes NS1-1, NS1-32 and NS1-34 were expressed and tested by ELISA, no positive reaction with MAbs and mouse sera against JEV NS1 was found for the homologous sequences from WNV and MVEV (Fig. 4, left panels). Further, no epitope-specific MAbs could recognize the corresponding peptides from WNV epitopes.

**Figure 5:**
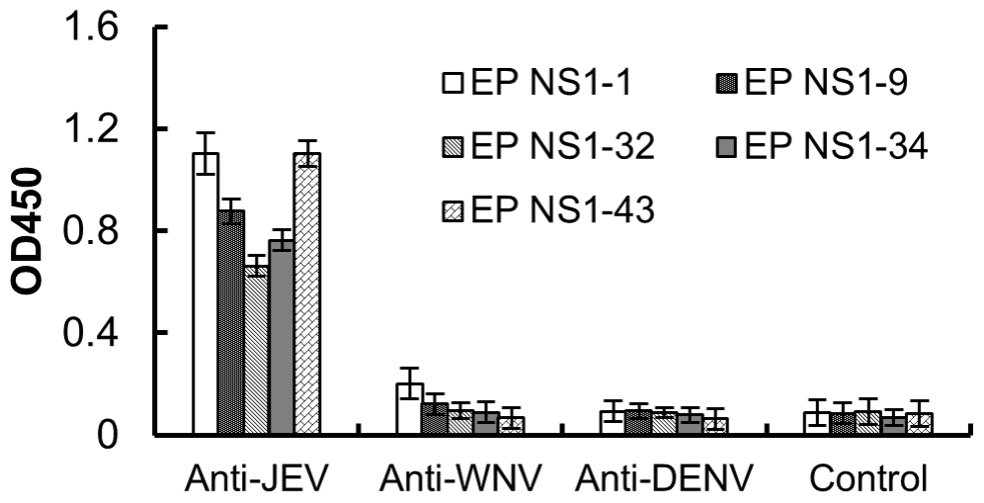
The JEV NS epitopes did not react with anti-WNV and anti-DENV sera. The identified epitope fusion proteins were used as coating antigen in an indirect ELISA. The reactivity of the epitope fusion proteins with anti-WNV and anti-DENV sera was assessed. Normal mouse serum was used as a control.

We then determined the reactivity of sera against WNV and DENV with the identified epitope-derived peptides. The results showed that neither sera against WNV nor DENV reacted with the five JEV NS1 epitopes (Fig. 5).

## Discussion

Flaviviral NS1 protein is involved in virus replication [25], [26] and induces a protective immune response against lethal virus infections though it does not induce neutralizing antibodies [27]. Passive administration of MAbs against NS1 or active immunization with NS1 gene/proteins confirmed protection from a lethal JEV challenge [5], [6], [28]. Serological cross-reactivity among members of the JEV serocomplex and other flaviviruses could confound detection results [29], especially in regions where two or more flaviviruses co-exist [30]. It was difficult to differentiate between viruses using ELISA or even by neutralization tests or other serological methods. The cross-reactivity among flaviviruses is primarily caused by the E glycoprotein [31]. Though NS1 is thought to contain more virus-specific epitopes than E protein [32], there are epitopes on NS1 of WNV cross-reacted with antibody against JEV-serocomplex virus [33]–[36]. Therefore, identification of epitopes on NS1 is important for subunit vaccine design and for developing serological tests that can differentiate between infections caused by flaviviruses of the JEV serocomplex and other flaviviruses.

In this study, we constructed a panel of JEV NS1-specific MAbs with 6×His-tagged and MBP-tagged recombinant JEV NS1 protein. After testing NS1-derived short fusion peptides against MAbs, five peptides were identified by eleven MAbs. These peptides correspond to JEV NS1 residues 1–16, 65–80, 249–264, 265–280 and 337–352, respectively, and are referred to as peptides NS1-1, -9, -32, -34 and -43. The peptides NS1-1, NS1-9 and NS1-32 were recognized by only one MAb; however, peptide NS1-34 was recognized by three MAbs, and peptide NS1-43 was recognized by five MAbs. Peptide NS1-34 and peptide NS1-43 may therefore contain more than one epitope respectively. In order finely map the epitopes, we designed and expressed sets of shortened fusion peptides for each of the above-mentioned peptides. After probing the shortened fusion peptides with MAbs, we confirmed five linear epitopes and deduced that the minimal units of epitopes of NS1-1, -9, -32, -34, -43 were ^5^AIDITRK^11^, ^72^RDELNVL^78^, ^251^KSKHNRREGY^260^, ^269^DENGIVLD^276^, ^341^DETTLVRS^348^, respectively.

Sequence alignment showed that the epitopes are highly conserved among JEV strains. Notably, epitopes NS1-9 and NS1-34 have 99% identity among 100 JEV strains, and there was only one amino acid substitution in all of the heterologous peptides corresponding to the respective epitope. The highly conserved nature of the epitopes we identified is beneficial for the application of epitopes in vaccine design and epitope-based diagnosis. We then compared the JEV NS1 protein sequence with the NS1 sequences of WNV, MVEV and DENV. Among the JEV-serocomplex, JEV has the highest NS1 sequence similarity with MVEV. JEV and DENV co-circulate in Southeast Asia [12], and JEV and WNV co-circulate in India [15], so there is an urgent need for differential diagnoses between JEV, WNV and DENV. None of the MAbs in this study reacted to WNV or DENV whole virus antigens or the corresponding NS1 sequences. Furthermore, none of the five JEV NS1 epitopes were recognized by anti-sera against WNV and DENV. Although the corresponding epitopes NS1-9 and NS1-43 of MVEV have weak reactivity with JEV NS1-specific MAbs and mouse antisera, there is no report of JEV and MVEV co-circulating in any region, and epitopes NS1-1, -32 and -34 did not cross-react with MVEV. This finding may indicate that these NS1 epitopes have the potential to be of use in serological monitoring and differential diagnosis.

In summary, we precisely identified five JEV-specific and highly conserved B-cell epitopes on JEV NS1. The data described in this work could provide a foundation for further understanding of the antigenic structure of NS1 and the potential clinical application of epitope-based methods.
